# Spatial-temporal attention for video-based assessment of intraoperative surgical skill

**DOI:** 10.1038/s41598-024-77176-1

**Published:** 2024-11-06

**Authors:** Bohua Wan, Michael Peven, Gregory Hager, Shameema Sikder, S. Swaroop Vedula

**Affiliations:** 1 Department of Computer Science, Whiting School of Engineering, Johns Hopkins University, Baltimore, 21218 USA; 2 Malone Center for Engineering in Healthcare, Johns Hopkins University, Baltimore, 21218 USA; 3 Wilmer Eye Institute, Johns Hopkins University School of Medicine, Baltimore, 21205 USA

**Keywords:** Surgery, Health care, Eye diseases, Lens diseases, Computational science, Computer science

## Abstract

Accurate, unbiased, and reproducible assessment of skill is a vital resource for surgeons throughout their career. The objective in this research is to develop and validate algorithms for video-based assessment of intraoperative surgical skill. Algorithms to classify surgical video into expert or novice categories provide a summative assessment of skill, which is useful for evaluating surgeons at discrete time points in their training or certification of surgeons. Using a spatial-temporal neural network architecture, we tested the hypothesis that explicit supervision of spatial attention supervised by instrument tip locations improves the algorithm’s generalizability to unseen dataset. The best performing model had an area under the receiver operating characteristic curve (AUC) of 0.88. Augmenting the network with supervision of spatial attention improved specificity of its predictions (with small changes in sensitivity and AUC) and led to improved measures of discrimination when tested with unseen dataset. Our findings show that explicit supervision of attention learned from images using instrument tip locations can improve performance of algorithms for objective video-based assessment of surgical skill.

## Introduction

Surgeons continuously acquire skill , specifically technical or psychomotor skill^1^and hereafter referred to as skill, throughout their careers^2–4^. Surgical skill affects patient outcomes, and poor skill is associated with an increased risk of severe adverse outcomes^5–8^. Surgeons early in their practice are more likely to cause intraoperative complications in their patients^9^. Assessment is critical to support the acquisition and maintenance of surgical skill through deliberate practice. Thus, assessment of surgical skill is necessary to protect public health through certification of competent surgeons.

Video-based assessment (VBA) is an effective method to assess intraoperative surgical technical skill. Videos of the surgical field capture a lot of information in the surgical field that is necessary to assess technical skill. Videos of the surgical field are easily available for a large number of surgical procedures that are performed with a camera, e.g., laparoscopic, endoscopic, microscopic, and robotic procedures. VBA has been a center-piece of recommended guidelines to assess surgical skill. VBA is potentially useful for professional certification of surgeons^10,11^, and it is an actively researched methodology to evaluate intraoperative surgical technical skill^12^.

VBA of surgical technical skill by expert surgeons is not efficient. Expert surgeon review takes a long time and it is susceptible to missing data^13^. Crowdsourcing is an alternative to expert review for VBA of surgical technical skill. Crowdsourcing allows rapid review of videos; however, the evidence on validity of crowdsourced ratings is still evolving. Early studies reported high correlations and small differences between crowdsourced and expert VBA ratings of surgical skill^14,15^. Evidence from previous study^13^ suggests poor correlation between crowdsourced and expert VBA ratings, and that direct expert observation or VBA is necessary.

Machine learning algorithms are an alternative to expert VBA^16^. Algorithms for VBA are efficient, provide reliable and objective assessments of surgical skill, and can be used on a large scale. Our research objective is to develop and validate algorithms for VBA of intraoperative surgical skill.

### Related work

Methods reported in prior studies for VBA of surgical skill in the operating room may be considered in three categories: 1. methods for end-to-end mapping of RGB video images to surgical skill; 2. methods that explicitly use structure in images, e.g., instrument motion; 3. methods that use localized contextual information about instruments, anatomy, or their interactions. Two approaches were used to train networks to extract spatial-temporal features to predict surgical skill using RGB videos of the surgical field from the operating room: 1. 3D-convolutional neural networks (CNNs); 2. CNN plus a recurrent neural network. Kitaguchi, D. et al^17^ used a 3D-CNN (Inception-V1 I3D) pretrained on the ImageNet and Kinetics datasets. The network was trained to classify video clips based upon scores on the Endoscopic Surgical Skill Qualification System for laparoscopic colorectal surgical procedures. On the other hand, Hira, S. et al^18^ used a CNN (ResNet) as an image encoder and a long short-term memory (LSTM) network to analyze the encoded images time series. The CNN-LSTM framework was trained to classify videos of a critical step in cataract surgery as expert or novice.

Unlike end-to-end networks, Lavanchy, J. L. et al^19^ proposed a 3-stage network for VBA of surgical skill. In the first stage, a CNN (ResNet-50 based feature pyramid network combined with faster R-CNN) is used to predict bounding boxes around instruments in the video images. The network was pre-trained on the Microsoft Common Objects in Context (COCO) dataset. A particle tracking algorithm was used to track instruments over time. In the second stage, several features were computed to describe motion of the instruments. In the third stage, the motion features were used to train a linear regression model to classify surgical skill (good vs. poor skill) in cholecystectomy. In a separate study, Hira, S. et al^18^ proposed a 2-stage network, in which the location of the tips of surgical instruments are predicted as keypoints using a network previously developed for human pose detection. The predicted instrument tip locations are then analyzed using a temporal convolutional network. The network was trained to classify surgical skill (expert vs. novice) for a critical step in cataract surgery.

The emphasis that the prior studies placed on instruments in the surgical field is no coincidence. Vedula, S. S. et al^16^ demonstrated the abundant evidence that instrument motion is highly informative of surgical skill. Hira, S. et al^18^ tested the hypothesis that training the network to learn where to focus both in the image (spatial) and in the time series (temporal) will improve algorithm performance. The CNN-LSTM architecture described above was enhanced with attention modules, one each for the spatial and temporal networks. The attention-based network had higher measures of discrimination than a network without attention modules.

**Fig. 1:**
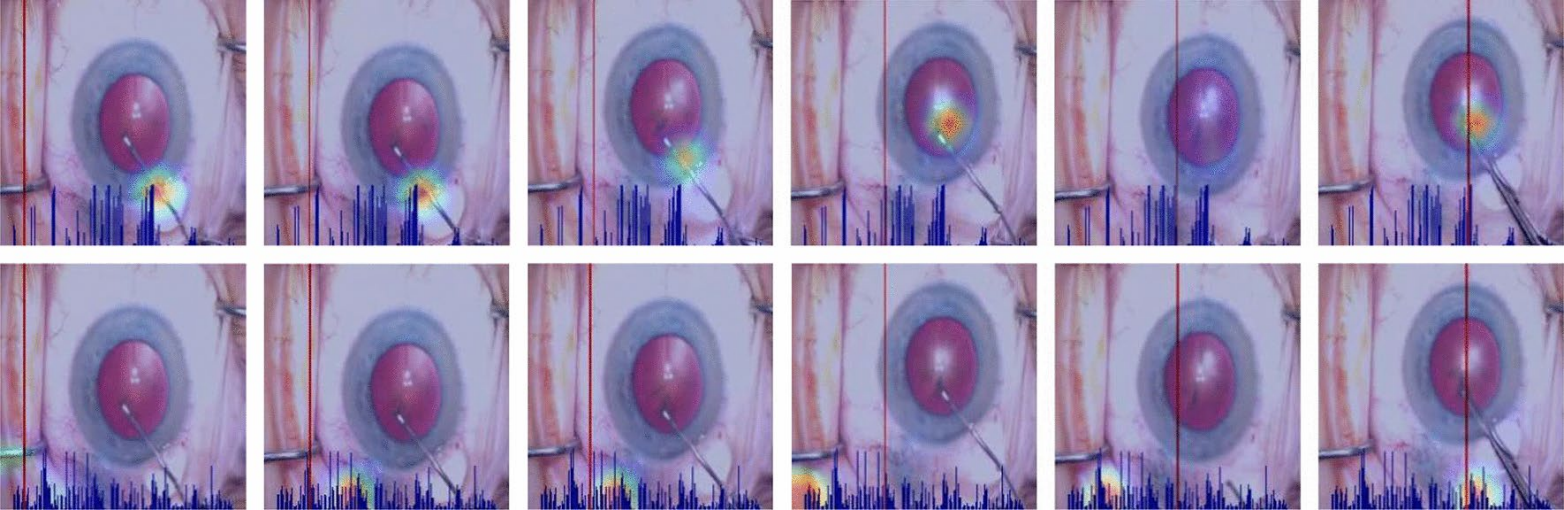
Comparison between supervised spatial attention map and unsupervised spatial attention map. The first row shows the supervised spatial attention maps. The attention maps are colored with the jet color map. The second row shows the unsupervised spatial attention maps. The bottom of each image shows the temporal attention map. The height of the blue bar denotes the attention weight of the corresponding frame. The red vertical line denotes the time location of the current frame. Note that there is no instrument in the fifth column and correspondingly, supervised attention (the first row) has low value on all image pixels while unsupervised attention (the second row) has high values around the bottom left corner. The temporal maps between top and bottom rows are different because they are generated from two separate models. The top row shows temporal maps generated from the model trained with supervised spatial attention, while the bottom row shows temporal maps generated from the model trained with unsupervised spatial attention.

The attention modules in the work of Hira, S. et al^18^ were unsupervised (trained without labels). While this led to an improvement in algorithm performance in one dataset, attention computed without supervision is not guaranteed to focus on relevant regions in the video, as illustrated in Fig. 1. As a result, the model may not have a similar performance in a new dataset (generalizability or external validity). Our objective in this work is to test the hypothesis that explicitly supervising attention improves network performance and generalizability for VBA of surgical technical skill. We expect that supervising spatial attention would allow the network to focus on the most informative regions in the images and improve the robustness against spurious features from irrelevant regions. Thus, it addresses the challenges posed by dataset shifts to generalizability of the algorithms. Our contributions in this work include:

1. explicit supervision of spatial attention using instrument tip locations in video images;
2. evaluation of two different approaches to compute spatial attention;
3. evaluation of supervised spatial attention using multi-task learning (to predict instrument tip locations as an auxiliary task) as a baseline multi-task learning is adopted as an additional baseline as it takes advantage of instrument tip locations. Comparing multi-task learning with supervised spatial attention shows the benefit of explicit supervision of spatial attention rather than implicit supervision using multi-task learning;
4. evaluation of the impact of spatial and temporal attention on model performance;
5. evaluation of different network architectures in comparison to a baseline;
6. evaluation of supervised spatial attention on both internal validity and external validity (or generalizability) of the networks. To evaluate generalizability, we used a held-out dataset separate from the training dataset.

## Method

In this section, we first describe the supervised spatial attention approach for VBA of surgical skill. Supervised attention assigns importance weightings to specific locations in a given feature map, an improvement over the traditional approach of global average pooling, which simply averages the features. Specifically, a feature map is first extracted from a frame using a pre-trained CNN backbone (ResNet-50 pretrained on ImageNet^20^, the use of which is a typical transfer learning approach, and it has been shown that ImageNet pretrained ResNet is generalizable^21,22^). Next, an attention map is produced through a linear transformation of the feature map to a matrix of scores indicating the importance of the corresponding region. The linear transform is supervised such that only those features that are located near the instrument are transformed to a high score. We propose two approaches for attending to the image feature using this computed attention map. The resulting feature is then used for the downstream task of skill classification.

This approach is based upon the assumption that an image feature map is provided for each frame of the surgical video. The feature map (and an optional temporal feature) are used to produce an attention map that attends to the image feature map to create an image feature vector. The process is supervised such that areas that are most informative about skill, in this case, near manually annotated instrument tip locations, have the highest values in the produced attention map. The details about the method of supervision are provided in the following sections. In Section 2.3 we introduce the formulation of unsupervised temporal attention adopted in this work. Finally, in Section 2.4 we apply supervised spatial attention using existing networks for VBA of surgical skill on a video dataset of cataract surgeries.

### Supervised spatial attention

**Fig. 2:**
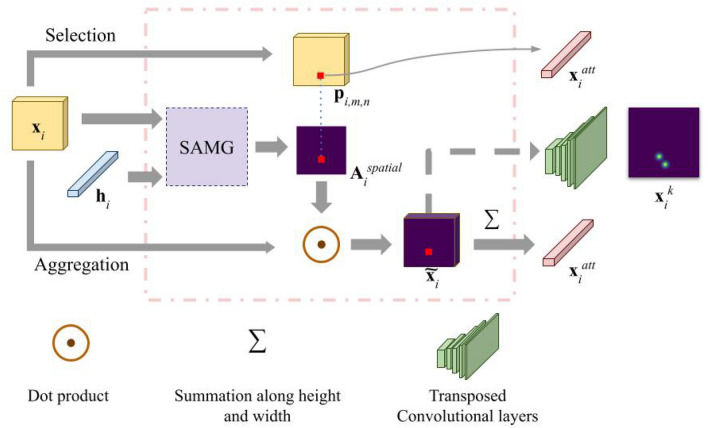
An illustration of the spatial attention module, outlined by the pink dashed box. The selection and aggregation scheme are denoted by the upper and lower streams, respectively. The two streams are mutually exclusive, and only one is chosen to use in practice. The temporal feature, ${\textbf{h}}_i$, is an optional input. The SAMG box denotes the process to compute the spatial attention map. $\bigodot$ is a dot product, and $\sum$ is a summation along the height and width dimensions of the attended feature map. The pathway of the multi-task learning model is denoted by the dashed arrow where the stacked green cubicles represent five layers of transposed convolutional layers. The area with the largest attention score in ${\textbf{A}}_i^{spatial}$ is used to localize image features for the downstream temporal model. This is represented by the blue dotted line.

A spatial attention map is calculated using image features of each frame and optionally, temporal features. The values in the spatial attention map indicate the importance of the corresponding regions in the image. As shown in Fig. 2, the spatial attention module takes two inputs. The first input is image feature vector (appearance feature) ${\textbf{x}}_i=\{{\textbf{p}}_{i,m,n}\}$ at $m \in [1,H], n \in [1,W]$ of the image feature map, where *H*, *W*are the height and width of the CNN backbone (ResNet-50 pretrained on ImageNet^20^) extracted image features, and frame $i \in [1,N]$. The second optional input is the LSTM hidden state (spatio-temporal features) ${\textbf{h}}_i$ at that frame. The score for the overall attention map ${\textbf{A}}_i^{spatial}$ for each region on the image feature map is calculated as follows:

1 $$\begin{aligned} f_{\text {appearance}}&= {\textbf{M}}_a{\textbf{p}}_{i,m,n} \end{aligned}$$

2 $$\begin{aligned} f_{\text {spatio-temporal}}&= {\textbf{M}}_s{\textbf{h}}_i \end{aligned}$$

3 $$\begin{aligned} f^{\text {overall}}_{m,n}&= {\textbf{M}}_o\sigma (f_{\text {appearance}} + f_{\text {spatio-temporal}}) \end{aligned}$$

4 $$\begin{aligned} att_{m,n}^{spatial}&= \frac{\exp (f^{\text {overall}}_{m,n})}{\sum _{m,n}\exp (f^{\text {overall}}_{m,n})} \end{aligned}$$

where $\sigma$is the ReLU (rectified linear unit) activation function^23^. To ensure compatibility of the operations, ${\textbf{M}}_a$, ${\textbf{M}}_s$, and ${\textbf{M}}_o$ are learned weight matrices for the appearance, spatio-temporal, and overall feature maps, respectively. Equation 2 and the $f_{spatio-temporal}$ term in equation 3 can be removed if temporal features are not available. Equation 1 to 4 first linearly transform image feature vectors ${\textbf{p}}_{i,m,n}$ to $f_{appearance}$ and optionally hidden state ${\textbf{h}}_i$ from the LSTM to $f_{spatial-temporal}$. Then, the results are activated with $\sigma$ (ReLU) and linearly transformed to the final feature $f^{overall}_{m,n}$ used for computing the spatial attention. Finally, equation 4 normalizes the features using softmax and produces the spatial attention.

Conventional attention models^24,25^ learn attention maps with task-oriented loss (e.g. cross-entropy loss). These attention maps represent a layer of re-weighting or “attending to” the image features. As we demonstrate in Fig.1, without explicit supervision, they are not guaranteed to localize relevant regions in the images. Furthermore, without a large amount of training data, attention mechanisms could assign higher weights to regions having spurious correlations with the target label^26,27^. Therefore, we hypothesize that explicitly supervising the attention map with relevant information in the images can improve both the accuracy of predictions and generalizability of the model.

Based on the previous work^16,28^, which demonstrates that instrument tip trajectories are highly informative of surgical skill, we construct binary trajectory heat maps ${\textbf{B}}_i$ for each frame *i*, combining the locations $s_{k,m,n}$ of all instrument tips, where *s* is a binary indicator variable denoting if instrument tip *k* is located within the receptive field of the region at coordinates *m*, *n*:

5 $$\begin{aligned} \forall b_{m,n}\in {\textbf{B}}_i, \quad b_{m,n}&={\left\{ \begin{array}{ll} 1 & \textit{ if } \sum \limits _k s_{k,m,n} \ge 1\\ 0 & \textit{otherwise} \end{array}\right. } \end{aligned}$$

For training, the overall loss function combines binary cross-entropy for skill classification $L_{BCE}$and the Dice coefficient^29^ between the spatial attention map ${\textbf{A}}^{spatial}$ and the tool-tip heat map ${\textbf{B}}$:

6 $$\begin{aligned} L_{Dice}&= DL(\{{\textbf{A}}_i^{spatial}|i\in [1,N]\},\{{\textbf{B}}_i|i\in [1,N]\}) \end{aligned}$$

7 $$\begin{aligned} L&= L_{BCE} + \lambda \cdot L_{Dice} \end{aligned}$$

The weighting factor $\lambda$ is empirically set to 0.5.

#### Aggregation

We propose two methods to attend to the image features. These methods summarize the image features using information from the computed attention map.

The first method of attending to the image features follows the aggregation scheme, where the attention-weighted image features $\widetilde{{\textbf{x}}}_i$ are summed over all pixels in each frame *i*:

8 $$\begin{aligned} {\textbf{x}}_{i}^{att}&= \sum \widetilde{{\textbf{x}}}_i = \sum {\textbf{A}}^{spatial}_{i}\cdot {\textbf{x}}_{i} \end{aligned}$$

The attention map ${\textbf{A}}_i^{spatial}$ is supervised using the trajectory heat map, biasing the attended image feature vector to have higher weights on features around the instrument tip.

#### Selection

The second way of attending on the image features follows the selection scheme. The attended image feature vector ${\textbf{x}}_i^{att}$ is computed as:

9 $$\begin{aligned} {\hat{m}},{\hat{n}}&=\mathop {\mathrm {arg\,max}}\limits _{m,n}(\{a_{m,n}^{spatial}|a_{m,n}^{spatial}\in {\textbf{A}}_i^{spatial}\}),\end{aligned}$$

10 $$\begin{aligned} {\textbf{x}}_{i}^{att}&= {\textbf{p}}_{{\hat{m}},{\hat{n}}} \end{aligned}$$

The selection scheme determines a region in the image feature map in eq. 9, leading to more localized features than the aggregation scheme. This approach is similar to masking the image using a detected bounding box, but it avoids the need for a separate detection network. Furthermore, using only a single region from the image feature map introduces regularization that helps prevent the network from overfitting. Our external validation experiments show that using the selection scheme yields significantly better results when tested on an external dataset.

### Multi-task learning baseline model

Multi-task learning is a technique that trains a network to perform multiple tasks at the same time. It exploits the commonalities and differences between tasks and usually results in improved performances. It is an intuitive baseline approach to compare a model with supervised attention maps as both utilize the information from instrument trajectories. In this approach, the network is trained to localize keypoints, i.e., predict the location of the instrument tips in the image. This involves adding a keypoint localization branch to the network (Fig. 2) and an additional loss term. The branch is implemented using five layers of transposed convolutional layers to up-sample the attention weighted image features $\widetilde{{\textbf{x}}_i}$to output keypoint predictions (as a heat map). We use the method proposed by Newell, A. et al^30^ to compute ground truth Gaussian heat maps using keypoint annotations and mean squared error loss during training.

### Unsupervised temporal attention

**Fig. 3:**
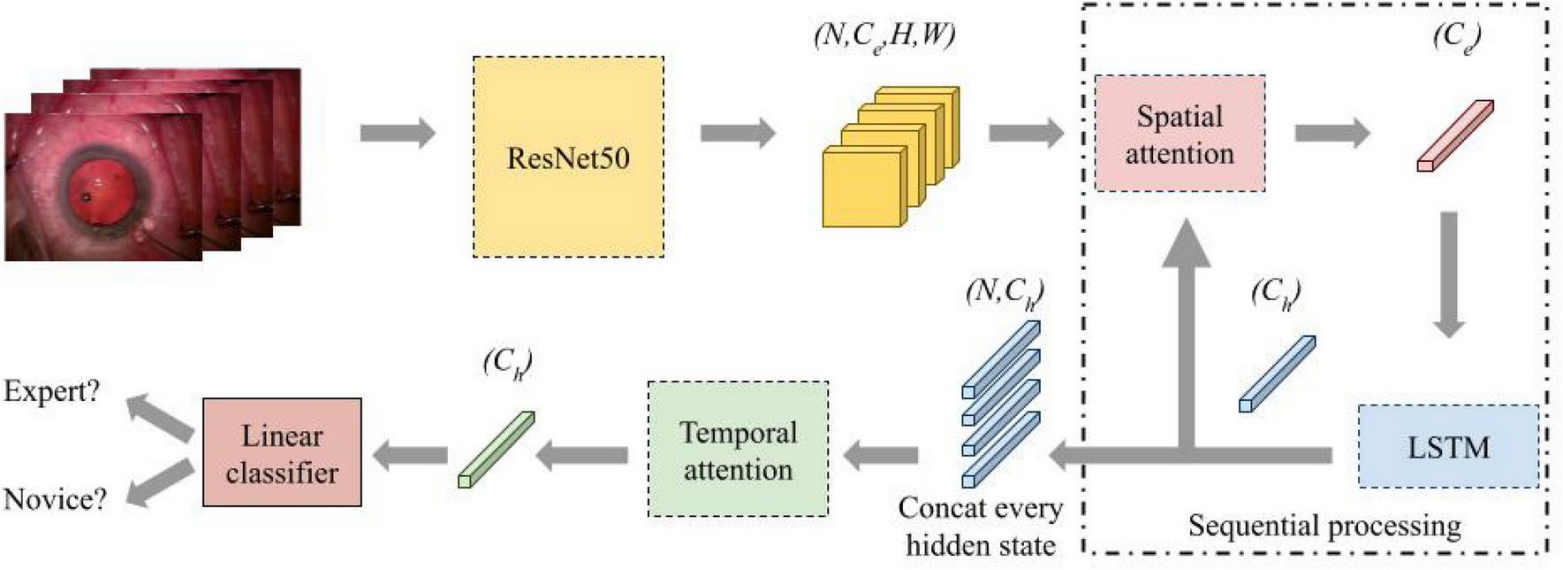
An overview of the CNN-LSTM network. The “Spatial attention” box is further illustrated in Fig. 2 and discussed in section 2.1. The “Temporal attention” box is discussed in section 2.3. $C_e$ and $C_h$ are the lengths of feature dimension of the image feature and the hidden state, respectively.

The last part of the model is a temporal attention mechanism as can be seen from Fig. 3. This component is used to aggregate all the hidden states from the LSTM to output a final feature for classification, and allows the network to learn which frame is more important to the final decision. The temporal attention ${\textbf{A}}^{temp}$ is calculated from the hidden states of the LSTM, ${\textbf{H}}=\{{\textbf{h}}_1,{\textbf{h}}_2,...,{\textbf{h}}_N\}$ as follows:

11 $$\begin{aligned} \mathbf {\beta }&= {\textbf{H}}{\textbf{h}}_N \end{aligned}$$

12 $$\begin{aligned} \forall i\in [1,N],\quad {\textbf{A}}^{temp}_i&= \frac{exp(\mathbf {\beta }_i)}{\sum _{i\in [1,N]}exp(\mathbf {\beta }_i)} \end{aligned}$$

13 $$\begin{aligned} {\textbf{h}}_f&=({{\textbf{A}}^{temp}})^T{\textbf{H}} \end{aligned}$$

Equation 11 first calculates dot product distance^31^, $\mathbf {\beta }$, between the final hidden state and all the hidden states from the LSTM. Then equation 12 normalizes the distance using softmax and produces the temporal attention ${\textbf{A}}^{temp}_i$. The attended final hidden state, ${\textbf{h}}_f$, is used as input to a linear model for classification. $\mathbf {\beta }$ is calculated using batched matrix multiplication to compute alignment between the hidden state at each time point, ${\textbf{H}}$, and the final hidden state of the LSTM, ${\textbf{h}}_N$.

### Integration with networks

1. CNN-LSTM proposed by Hira, S. et al^18^. Fig. 3 shows the structure of the integration of our method and the baseline network;
2. CNN-GRU. This baseline is similar to the CNN-LSTM, except that we replaced the LSTM module in^18^’s network with the GRU (gated recurrent units) module;
3. CNN-Transformer. This baseline is also modified from the CNN-LSTM network. We replaced the LSTM module with the transformer^31^ and removed

the hidden state input (temporal feature) to the “Spatial attention” box in Fig. 3. For more details, see Section 2.1 on how to generate the spatial attention map without temporal features. The temporal attention is removed as a transformer is already a temporal attention model.

### No attention network

**Fig. 4:**
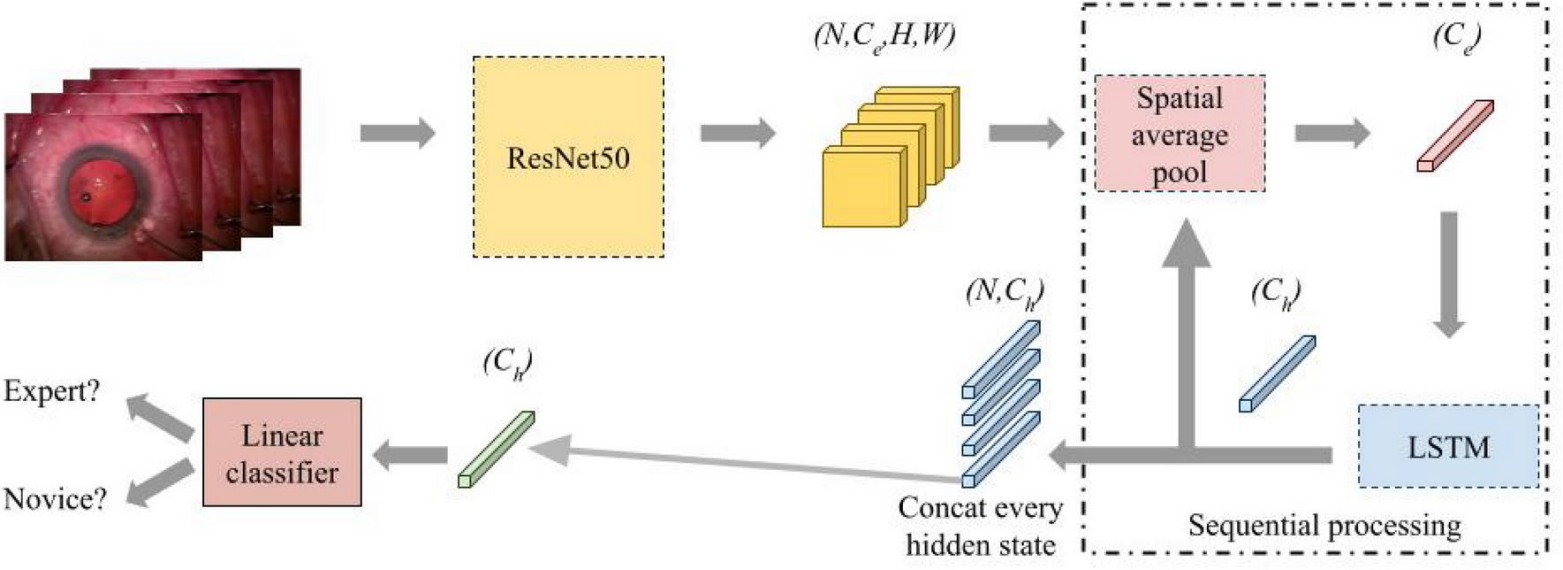
The network architecture of the no attention model.

To evaluate the effect of supervised spatial attention and temporal attention modules, we design and implement a no attention network, whose structure is shown in Fig. 4. The no attention network is modified from the CNN-LSTM network. The spatial attention module is replaced with average pooling and the hidden state of the last frame is used as the final classification vector.

## Experiments and discussion

All analyses reported in this study were performed in accordance with the relevant regulations and requirements. The Institutional Review Board at Johns Hopkins Medical Institutions approved the research reported in this manuscript. Informed consent was obtained from the study participants.

### Dataset

#### Source dataset

We use the dataset described in previous work^18,28^ as the source dataset. The dataset contains 99 videos of capsulorhexis, a critical step in cataract surgery. The videos were captured from the operating microscope, and processed to have a resolution of 640*480 and a frame rate of 59 frames per second. An expert surgeon , a faculty at a tertiary care academic center with more than 10 years of experience, assigned ground truth ratings of skill using the International Council of Ophthalmology’s Ophthalmology Surgical Competency Assessment Rubric-Phacoemulsification (ICO-OSCAR:phaco)^32^. Using the scores on the two items for capsulorhexis in ICO-OSCAR:phaco, which are rated on a Likert scale ranging from 2 to 5, we assigned videos an expert label if the score on at least one of the items was a 5 and the score on the other item was at least a 4. The video was assigned a novice label if these criteria were not met. The dataset includes 51 expert and 48 novice videos, which were evenly distributed among five folds for cross-validation. One individual, without medical education but trained to annotate, manually marked the location of instrument tips and the points of entry of instruments into the eye for every twelfth frame in each video. The annotation was done by clicking on the image displayed on the screen. The location of the click was recorded using a program written in Matlab (Mathworks, Inc., Natick, USA).

#### Target dataset

The target dataset includes 51 videos from surgeries performed in the same institution as the source dataset but a few years later. The recording process, frame rate, resolution, and skill annotation methodology were identical to that for the 99 videos dataset. However, the target dataset did not include annotations on instrument tip locations. In addition, the target dataset includes five expert videos and 46 novice videos. We use this dataset for external validation (target domain).

#### Comparison between the source dataset and the target dataset

**Fig. 5:**
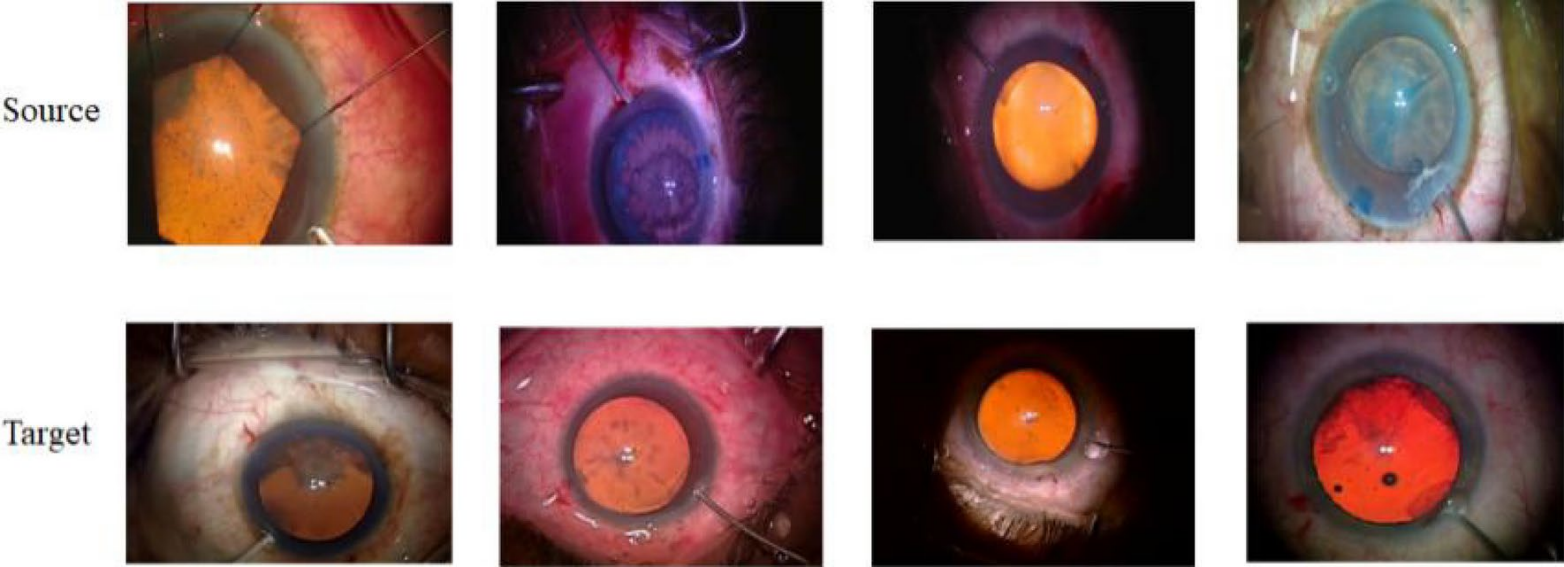
Images sampled from the source dataset and the target dataset.

Fig. 5 shows some sample images of the source and target datasets.

**Table 1:** Statistics of the lengths of the videos (number of frames) from the source dataset and the target dataset.

	Mean	Std	Min	Max
Source	8677	6208	1712	29979
Target	20790	17080	5162	83389

Table 1shows there is a significant covariate shift^33^between the two datasets. The mean, minimum, and maximum length of the videos from the target dataset are almost three times longer than those in the source dataset. Furthermore, the standard deviation of the videos in the target dataset is also almost three times larger than those in the source dataset. Moreover, videos from the target dataset show procedures performed by residents, who are trainees. Videos from the source dataset are performed by expert surgeons and residents. Movements by expert surgeons tend to be more efficient and smooth than those by novices^34^. The differences in the speed of movements and lengths of the videos between the two datasets are the main contributors to the covariate shift.

**Table 2:** Class label distribution statistics for the source and the target datasets.

	Number of videos	Expert	Novice	Operating surgeons
Source	99	50	49	Expert surgeons and residents.
Target	51	5	46	Residents only.

Table 2 shows that there is also a significant label shift between the two datasets. The target dataset is scarce in positive samples. This could make domain adaptation especially difficult, as there might not be sufficient positive samples from the target domain to include in the source domain for semi-supervised domain adaptation.

### Data processing

In this section, we introduce the sampling strategy used in this work and the data augmentation techniques applied.

#### Sampling

We sampled a small portion of the video while preserving most of the information to accommodate computational limitations and redundancy between consecutive frames. Following the sampling strategy used by Hira, S. et al^18^, a clip of 256 frames is sampled for each video. First, we uniformly and randomly sample a starting frame. Next, we select 256 frames, subsampling every 8th frame. This strategy will preserve most of the information and cover of the whole video after a sufficient amount of repetitions. Setting the sampling interval as 8 will ensure minimal loss of information in the time window of 256 frames. Since we are randomly selecting the starting frame, each sampled clip will be different. Furthermore, we sample a different clip from the video for each training epoch. Thus, with a sufficient amount of training epochs, all the sampled clips are expected to sparsely cover the whole video. During testing, we sample three times and test the model with the three sampled clips. We average the results of the three clips as the final result. The purpose of multiple sampling is that each sample only contains 256 frames, which is only a small portion of the whole video. During testing, sampling three times increases the exposure of the video to the algorithm while maintaining a low computational cost.

#### Data augmentation

Data augmentation is a useful technique to increase the size and diversity of the training data. Both our source dataset and target dataset are small in size, making data augmentation even more important. We use random crop, color jittering, horizontal flip, and random rotation to augment frames from the videos. For the source dataset, the instrument tip locations are augmented with the corresponding images. We use the Albumentation 1.01 framework^35^ for the augmentations.

### Implementation details

**Table 3:** A list of models implemented for our experiments.

No.	Model	Temporal Module	Temporal Attention	Supervised	Spatial Attention
1	$\hbox {LSTM}_{{agg}}$	LSTM	✓	✗	Aggregation
2	LSTM-$\hbox {MUL}_{{agg}}$	LSTM	✓	✗	Aggregation
3	$\hbox {LSTM}_{agg}^s$	LSTM	✓	✓	Aggregation
4	$\hbox {LSTM}_{sel}^s$	LSTM	✓	✓	Selection
5	$\hbox {LSTM}_{agg}^{s-t}$	LSTM	✗	✓	Aggregation
6	$\hbox {LSTM}_{sel}^{s-t}$	LSTM	✗	✓	Selection
7	$\hbox {LSTM}_{no\_spatial}$	LSTM	✓	✗	None
8	$\hbox {LSTM}_{no\_spatial}^{-t}$	LSTM	✗	✗	None
9	$\hbox {GRU}_{{agg}}$	GRU	✓	✗	Aggregation
10	$\hbox {GRU}_{agg}^s$	GRU	✓	✓	Aggregation
11	$\hbox {GRU}_{sel}^s$	GRU	✓	✓	Selection
12	$\hbox {Transformer}_{{agg}}$	Transformer	✓	✗	Aggregation
13	$\hbox {Transformer}_{agg}^s$	Transformer	✓	✓	Aggregation
14	$\hbox {Transformer}_{sel}^s$	Transformer	✓	✓	Selection

Model No. 1 :  We reproduced the spatial temporal attention CNN-LSTM model presented by^18^ and use it as the baseline model.

Model No. 2 :  The LSTM-$\hbox {MUL}_{{agg}}$ is the $\hbox {LSTM}_{{agg}}$ model with multi-task learning as discussed in section 2.2.

$^{s}:$ This superscript means the spatial attention in this model is supervised with the instrument tip locations.

$^{-t}:$ This superscript means the temporal attention module is removed from the model.

$_{no\_spatial}:$ This subscript means the spatial attention model is removed from the model.

The number in the first column (No.) is used to reference the corresponding model in the following text.

**Frameworks **We use the PyTorch 1.3 framework^36^ for implementation, training, and evaluation.

**Models** We implemented a total of 14 models for our experiments. Table 3 shows the specification of each model.

**Training **We used the Adam optimizer with an initial learning rate of 1e-3 and decreased it by a factor of 10 as validation loss plateaued. The batch size is set as 2 for all models except for the multi-task learning model, for which it was set to 1 due to computational constraints. We used a ResNet-50 backbone, which was pre-trained on ImageNet^20^, and froze its weights when training our models. However, when training our models with the selection scheme, we unfreeze the last block of the ResNet-50 backbone. We set the $C_{e}$ as 2048 and $C_h$ as 1024. The dimensions of ${\textbf{M}}_{e}$, ${\textbf{M}}_p$, and ${\textbf{M}}_{h}$, are $(1024\times 1)$, $(1024\times 1024)$, and $(2048\times 1024)$, respectively. The final linear classification layer (dimensions = (1024, 1)) is followed by the sigmoid function. All hyper-parameters are empirically tuned.

**Evaluation **We evaluated the models for both internal and external validity. Internal validation means that the training, validation, and test data are drawn from the same dataset, namely the source dataset. External validation means the training and validation data are drawn from the source dataset and the test data are drawn from the target dataset^37,38^.

For experiments, we used the same 5-fold cross-validation setup described in previous work^18^. To evaluate the models’ robustness against multiple forms of dataset shift (see 3.1), we train our models on the source dataset using 5-fold cross validation and then test on the target dataset with the best model (with the lowest validation loss). We computed the estimates and 95% confidence intervals of accuracy, sensitivity, specificity, and AUC to evaluate model performance. Sensitivity is defined as $\frac{TP}{TP+FN}$, where *TP* stands for the number of correctly classified positive samples, and *FN* stands for the number of positive samples that are incorrectly classified as negative. Sensitivity measures of all the positive samples, how many are classified as positive. Specificity is defined as $\frac{TN}{TN+FP}$, *TN* represents the number of negative samples that are correctly classified as negative, and *FP* stands for the number of negative samples that are incorrectly classified as positive. Specificity measures of all the negative samples, how many are classified as negative. AUC is the area under the receiver operating characteristic curve (ROC). The ROC is a plot of sensitivity versus 1-specificity at various thresholds for binary classification. In binary classification, the model outputs a probability of the sample being positive. If the probability is greater than the threshold, which is usually 0.5, then the sample is classified as positive, and otherwise, negative. The ROC allows measuring how well the model performs using various thresholds. The AUC may be interpreted as the probability that the model correctly outputs a higher probability of being positive for an expert video than for a novice video in any pair of videos sampled from the dataset.

### Results

#### Baseline models

We replicated the findings reported in Hira, S. et al’s work^18^ using an identical network architecture, referred to as $\hbox {LSTM}_{{agg}}$ (model No. 1). Differences in estimates (higher AUC, and lower sensitivity and specificity) are due to the inherent stochasticity in training and using a larger batch size. Multi-task learning model discussed in section 2.2 is another baseline model.

It’s important to note that we are not using the prior work of Hira, S. et al^18^ and Kim, T. S. et al^28^ to substantiate our own; rather, we take inspiration from their idea that instrument usage contains information necessary to assess skill. We develop an alternative method for using instrument tip locations, along the lines of recent developments in the broader machine learning community of using supervised attention^27,39^. We compared our results with those reported in Hira, S. et al’s work^18^ to show an improvement in performance. Furthermore, previous studies do not provide any other baselines against which we can compare our approach to supervised attention. The majority of previous research on VBA of surgical skill used datasets from surgical simulation^40^. The few studies that used surgical videos from the operating room did not evaluate supervised attention^19,41^. For this reason, we rely on Hira, S. et al’s model^18^ as a baseline model for comparison, which is also the previous state-of-the-art on the source dataset we used in this study. Additionally, Hira, S. et al^18^ do not perform external validation on a separate target dataset, which is an important analysis to determine whether the algorithm can be used in practice.

#### Effect of supervising spatial attention

**Table 4:** Comparison of performance of models with supervised spatial attention and baseline models.

No.	Model	Internal			External		
		Sensitivity	Specificity	AUC	Sensitivity	Specificity	AUC
1	$\hbox {LSTM}_{{agg}}$	**0.78** (0.65 to 0.88)	0.71 (0.57 to 0.82)	0.82 (0.73 to 0.91)	0.60 (0.23 to 0.88)	0.09 (0.03 to 0.20)	0.37 (0.06 to 0.68)
2	LSTM-$\hbox {MUL}_{{agg}}$	0.67 (0.53 to 0.78)	0.73 (0.59 to 0.83)	0.73 (0.63 to 0.83)	-	-	-
3	$\hbox {LSTM}_{agg}^s$	0.76 (0.63 to 0.86)	0.79 (0.66 to 0.88)	**0.85** (0.77 to 0.93)	0.07 (0.02 to 0.18)	**1.00** (0.57 to 1.00)	**0.50** (0.13 to 0.88)
4	$\hbox {LSTM}_{sel}^s$	0.73 (0.59 to 0.83)	**0.81** (0.68 to 0.90)	**0.85** (0.78 to 0.93)	0.40 (0.12 to 0.77)	0.41 (0.28 to 0.56)	0.45 (0.23 to 0.67)

Model No. 1 :  We reproduced the spatial temporal attention CNN-LSTM model presented by^18^ and use it as the baseline model.

Model No. 2 :  The LSTM-$\hbox {MUL}_{{agg}}$ is the $\hbox {LSTM}_{{agg}}$ model with multi-task learning as discussed in section 2.2.

$^{s}:$ This superscription means the spatial attention in this model is supervised with the instrument tip locations.

The best result (results) in each column are in bold. 95% confidence interval are in parentheses below the estimate.

Augmenting the baseline model ($\hbox {LSTM}_{{agg}}$, model No. 1) with supervised spatial attention was associated with a considerable improvement in estimates of specificity in both internal and external validation (source and target), as shown in Table 4. The improvement of estimates of specificity could be the result of models being more focused on the area around the instrument, where the most informative information about the skill is shown. Poor skills are often shown around the instrument in the form of diffident wielding of the instrument and damaging of the anatomy. Such patterns can be captured locally in frames and more easily recognized by the models if they are supervised to focus on those areas around the instrument. Recognizing highly skilled surgeons from the videos requires better reasoning of global features. Such global reasoning is more related to the temporal modeling component of the models than the spatial attention component, which could explain why the estimates of sensitivity of the models do not benefit from supervised spatial attention. Supervision of spatial attention during training appears to improve generalizability of the algorithms even when test data does not include the signal used for supervision (i.e., instrument tip locations in images). Compared with the baseline model with no supervision of spatial attention ($\hbox {LSTM}_{{agg}}$, model No. 1), supervision was associated with a considerable improvement in one or more estimates of measures of discrimination in the external validation experiments. Using the selection scheme to attend to image features ($\hbox {LSTM}_{sel}^s$, model No. 4) led to the most meaningful improvement in performance despite lower sensitivity than the baseline model. It is intuitive that the selection scheme led to more robust improvements compared with the aggregation scheme because the selection scheme uses a single feature vector from the region in the spatial feature map with the highest attention, which is supervised to be the region that contains the most relevant information to skill, while the aggregation scheme is essentially a weighted summation of the features of all the regions and thus, irrelevant information is retained for downstream processing. On the other hand, supervision and the aggregation scheme ($\hbox {LSTM}_{agg}^s$, model No.3) led to an improvement in specificity but a large drop in sensitivity.

#### Ablation studies

**Table 5:** Ablation study on spatial and temporal attention. Models with or without temporal attention and supervised spatial attention are compared.

No.	Model	Internal			External		
		Sensitivity	Specificity	AUC	Sensitivity	Specificity	AUC
3	$\hbox {LSTM}_{agg}^s$	0.76 (0.63 to 0.86)	0.79 (0.66 to 0.88)	0.85 (0.77 to 0.93)	0.07 (0.02 to 0.18)	**1.00** (0.57 to 1.00)	0.50 (0.13 to 0.88)
4	$\hbox {LSTM}_{sel}^s$	0.73 (0.59 to 0.83)	0.81 (0.68 to 0.90)	0.85 (0.78 to 0.93)	0.40 (0.12 to 0.77)	0.41 (0.28 to 0.56)	0.45 (0.23 to 0.67)
5	$\hbox {LSTM}_{agg}^{s-t}$	**0.78** (0.65 to 0.88)	**0.83** (0.70 to 0.91)	**0.88** (0.81 to 0.95)	0.40 (0.12 to 0.77)	0.80 (0.67 to 0.89)	0.60 (0.34 to 0.87)
6	$\hbox {LSTM}_{sel}^{s-t}$	0.61 (0.47 to 0.73)	0.75 (0.61 to 0.85)	0.77 (0.67 to 0.86)	**0.60** (0.23 to 0.88)	0.57 (0.42 to 0.70)	**0.67** (0.51 to 0.82)
7	$\hbox {LSTM}_{no\_spatial}$	**0.78** (0.65 to 0.88)	0.79 (0.66 to 0.88)	**0.88** (0.81 to 0.95)	**0.60** (0.23 to 0.88)	0.43 (0.30 to 0.58)	0.50 (0.26 to 0.73)
8	$\hbox {LSTM}_{no\_spatial}^{-t}$	**0.78** (0.65 to 0.88)	** 0.83** (0.70 to 0.91))	0.87 (0.80 to 0.94)	**0.60** (0.23 to 0.88)	0.35 (0.23 to 0.49)	0.47 (0.11 to 0.83)

$^{s}:$ This superscription means the spatial attention in this model is supervised with the instrument tip locations.

$^{-t}:$ This superscription means the temporal attention module is removed from the model.

The best result (results) in each column are in bold. 95% confidence interval are in parentheses below the estimate. Results of model No. 3 and No. 4 are repeated from table 4 to make for easy reading.

We further evaluated the effect of spatial attention and temporal attention in the CNN and LSTM network architecture in an ablative experiment. Results are presented in table 5. When validated internally, omitting spatial attention alone ($\hbox {LSTM}_{no\_spatial}$, model No. 7) leads to negligible changes in AUC and specificity compared to supervising spatial attention ($\hbox {LSTM}_{agg}^s$, model No. 3, and $\hbox {LSTM}_{sel}^s$, model No. 4, whose results are shown in table 4), and a small improvement in sensitivity, which is larger for $\hbox {LSTM}_{sel}^s$ than $\hbox {LSTM}_{agg}^s$. In external validation experiments, omitting spatial attention alone ($\hbox {LSTM}_{no\_spatial}$) was associated with negligible changes in AUC, significant improvement in sensitivity compared to supervised attention models ($\hbox {LSTM}_{sel}^s$ and $\hbox {LSTM}_{agg}^s$). There is a significant drop in specificity when compared to $\hbox {LSTM}_{agg}^s$ but less meaningful as the sensitivity is close to 0. From these results, it appears that with temporal attention, with or without supervised spatial attention the model performs similarly or even worse in terms of sensitivity. However, when not using temporal attention, omitting spatial attention was associated with a loss in generalizability. In external validation, the estimate of AUC and specificity of $\hbox {LSTM}_{no_spatial}^{-t}$ (model No. 8) are lower than that of $\hbox {LSTM}_{agg}^{s-t}$ (model No. 5) and $\hbox {LSTM}_{sel}^{s-t}$ (model No. 6). Furthermore, $\hbox {LSTM}_{agg}^{s-t}$ and $\hbox {LSTM}_{sel}^{s-t}$ achieved the top two estimates of AUC. These results show supervised spatial attention helps improve model generalizability when temporal attention is absent.

Omitting temporal attention was associated with a small improvement in estimates of AUC, sensitivity, and specificity when supervising spatial attention and attending to image features using the aggregation scheme ($\hbox {LSTM}_{agg}^s$, model No. 3, versus $\hbox {LSTM}_{agg}^{s-t}$, model No. 5) in internal validation. Similar results are found in external validation except $\hbox {LSTM}_{agg}^s$ had higher specificity. From our findings, it appears that temporal attention hinders the model’s performance when the spatial attention was supervised using the aggregation scheme. We hypothesize that it was because supervised spatial attention already selected the most important information in each frame and using unsupervised temporal attention may lead to overfitting. However in internal validation, estimates for all measures were considerably lower when omitting temporal attention but supervising spatial attention and attending to image features using the selection scheme ($\hbox {LSTM}_{sel}^s$, model No. 4, versus $\hbox {LSTM}_{sel}^{s-t}$, model No. 6). Since the selection scheme already excluded most information in each frame, removing temporal attention can cause the model to underfit the training data, leading to lower estimates of performance. Contrary to the aggregation scheme, the selection scheme introduces no additional parameters. Therefore, removing the temporal attention while using the selection scheme will result in a sever decrease in the amount of parameters. The only trainable component in $\hbox {LSTM}_{sel}^{s-t}$ is the LSTM component. Therefore, the model was most likely underfitting the training data and thus achieved lower estimate of AUC compared to $\hbox {LSTM}_{sel}^s$. However, fewer parameters would also help the model be more generalizable, and as expected, in the external validation experiments, the $\hbox {LSTM}_{sel}^{s-t}$ outperformed $\hbox {LSTM}_{sel}^s$ by a considerable margin in terms of the estimate of AUC.

Omitting temporal attention when spatial attention was not used to train the network results in negligible changes in estimates of algorithm performance ($\hbox {LSTM}_{no\_spatial}$, model No. 7, versus $\hbox {LSTM}_{no\_spatial}^{-t}$, model No. 8) in both internal and external validation. Moreover, they are outperformed by supervised spatial attention without temporal attention ($\hbox {LSTM}_{agg}^{s-t}$, model No. 5, and $\hbox {LSTM}_{sel}^{s-t}$, model No. 6) These results show it is unnecessary to use attention at all for our source dataset, where only a small amount of data is available and the data are evenly distributed and easy for the model to learn representative features. However, such representative features learned by no attention models are not robust against domain shifts.

#### Effect of different architectures

**Table 6:** Model performance with different temporal architectures. The default temporal architecture, LSTM, is replaced with either GRU or Transformer.

No.	Model	Internal			External		
		Sensitivity	Specificity	AUC	Sensitivity	Specificity	AUC
9	$\hbox {CNN}_{{agg}}$-GRU	0.75 (0.61 to 0.84)	0.83 (0.70 to 0.91)	0.84 (0.76 to 0.92)	**1.00** (0.57 to 1.00)	0.00 (0.00 to 0.08)	0.36 (0.16 to 0.56)
10	$\hbox {CNN}_{agg}^s$-GRU	0.76 (0.63 to 0.86)	0.83 (0.70 to 0.91)	0.85 (0.77 to 0.93)	0.60 (0.23 to 0.88)	**0.70** (0.55 to 0.81)	**0.67** (0.42 to 0.92)
11	$\hbox {CNN}_{sel}^s$-GRU	0.73 (0.59 to 0.83)	0.81 (0.68 to 0.90)	**0.86** (0.79 to 0.93)	0.80 (0.38 to 0.96)	0.39 (0.26 to 0.54)	0.63 (0.32 to 0.95)
12	$\hbox {CNN}_{{agg}}$-Transformer	**0.78** (0.65 to 0.88)	0.69 (0.55 to 0.80)	0.82 (0.74 to 0.90)	0.60 (0.23 to 0.88)	0.04 (0.01 to 0.15)	0.22 (0.02 to 0.42)
13	$\hbox {CNN}_{agg}^s$-Transformer	0.73 (0.59 to 0.83)	0.79 (0.66 to 0.88)	0.82 (0.73 to 0.90)	0.60 (0.23 to 0.88)	0.04 (0.01 to 0.15)	0.23 (0.00 to 0.47)
14	$\hbox {CNN}_{sel}^s$-Transformer	0.73 (0.59 to 0.83)	**0.87** (0.75 to 0.94)	0.83 (0.75 to 0.91)	0.60 (0.23 to 0.88)	0.43 (0.30 to 0.58)	0.60 (0.32 to 0.88)

$^{s}:$ This superscription means the spatial attention in this model is supervised with the instrument tip locations.

The best result (results) in each column are in bold. 95% confidence interval are in parentheses below the estimate.

As shown in table 6, replacing the LSTM with a GRU improved specificity with unsupervised spatial attention ($\hbox {GRU}_{{agg}}$) and supervised with the aggregation scheme ($\hbox {GRU}_{agg}^s$), but not when it was supervised and the selection scheme was used ($\hbox {GRU}_{sel}^s$) in internal validation experiments. On the other hand, replacing the LSTM with a transformer improved specificity only when using the selection scheme ($\hbox {Transformer}_{sel}^s$). Differences in estimates of AUC and sensitivity among the different architectures were small in magnitude.

In external validation experiments, replacing the LSTM with a GRU or a Transformer when spatial attention was not supervised did not yield any meaningful change in algorithm performance ($\hbox {LSTM}_{{agg}}$ versus $\hbox {GRU}_{{agg}}$ and $\hbox {Transformer}_{{agg}}$). Replacing the LSTM with a GRU led to meaningful changes in estimates when using the aggregation scheme to attend to image features ($\hbox {GRU}_{agg}^s$). On the other hand, using the selection scheme to attend to image features performed better than replacing the LSTM with a transformer ($\hbox {Transformer}_{sel}^s$).

Overall, supervising the spatial attention generally improves model’s performance in one or more measurements in internal or external validations. These results show our method is beneficial to different choices of temporal architecture. Our methods were not intended to solve the problem of generalizability problem. Our findings suggest that while supervising spatial attention is useful for generalizability of the algorithms, it is insufficient. Other approaches such as data augmentations in the temporal domain are necessary and should be evaluated in future research.

### Discussion

VBA of surgical technical skill using neural networks is a well studied problem with most research conducted using data from the simulation settings^16,42^. In videos of the surgical field, information necessary to assess skill is distributed across images and across time. However, information on surgical technical skill is not uniform across images and time. Across images^16^, showed that when using kinematics, the surgical end effector is a primary source of information on surgical skill. While there is limited research to localize surgical skill in the temporal domain, performance may vary across time either because of task difficulty, skill deficits, fatigue, or other reasons^5,43–45^.

Multi-task learning was proposed as a paradigm to maximize information around tips of the surgical instruments to assess skill. In datasets from surgical simulation, multi-task learning was used to train networks that were highly accurate for classifying surgical skill^46,47^. Our findings in this study suggest that the complexity in videos of the operating room means that information localized on instruments alone is not sufficient.

Attention mechanisms to emphasize localized information have been explored to develop networks for applications in surgical data science. Our prior work^18^ illustrates application of attention to surgical skill assessment. Others used attention to fuse multimodel sensors for better gesture recognition^48^. Different from our work, they adopted self-attention, which is still unsupervised. The learned attention in these works is not assured to focus on regions that are most informative for the prediction task because no constraints were placed on the attention module. Consequently, the risk of attending on irrelevant regions within the images is high. Model predictions when the attention is focused on irrelevant regions are not likely to generalize to new data. In addition, attending on irrelevant regions of the images will result in degradation of surgeons’ trust in the model’s decisions. To address limitations with unsupervised attention, our approach explicitly guides the network to focus on the regions within images that are most relevant to surgical skill assessment (which is around the instrument tips). Although our approach requires the acquisition of instrument tip locations, there are existing approaches for automated instrument tip locations acquisition from videos. Lavanchy, J. L. et al^19^ proposed a three-staged machine learning algorithm for automated skill assessment. In the first two steps of their approach instrument tip locations are obtained in a fully automatic manner. Future research may focus on evaluating the impact of using such automatically generated instrument tip locations versus to using manually annotated locations. Such automatic instrument tip localization algorithms would allow future work to build much larger datasets and better evaluate the generalizability of our approach and other models. Furthermore, research may also focus on supervising the spatial attention with other signals such as anatomies and study how to fuse multi-head attention where each head is supervised with different signals. It would also be natural to extend our work on supervised temporal attention. Our results show supervised spatial attention is more robust against domain shifts. It would be interesting to see if supervised temporal attention has a similar effect. Moreover, exploring how to effectively supervise attention generated with other mechanisms, such as self-attention, would be interesting.

The goal of objective VBA of surgical technical skill is to develop accurate, generalizable algorithms that provide explainable predictions. Our approach improved model performance and robustness against domain shift. Given that we used a small dataset, we don’t believe that the models we trained are ready for clinical use. The networks should be trained on a large dataset and evaluated using videos from many surgeons. However, there are no widely accepted benchmarks for accuracy or other metrics to show that the models can be used in the clinic. It is now a topic of active discussion in the surgical community^12^. We used a binary ground truth that corresponds to a summative assessment of surgical skill. There is consensus in the surgical community that VBA using algorithms should address summative assessment^49^. Some applications of summative assessments of surgical skill include evaluating trainees at discrete time intervals to monitor progress, certification and re-certification of surgeons^50^, and determining the operating privileges a surgeon can be provided. We derived the ground truth based on a widely accepted structured rating scale to assess skill in cataract surgery. Despite the limitations discussed here, our work is a step towards generalizable objective VBA of surgical skill system. Furthermore, our approach is explainable in that the supervised spatial attention map focuses on the most informative regions. Giving surgeons information on what information the network is using to reach a prediction would engender trust from the surgeons.

#### Limitations

Our work has the following limitations that should be addressed in future research. We used manually annotated and not predicted locations of instrument tips. However, our approach only needs the instrument tip location in the feature map space (generated from the ResNet-50). Therefore, as long as the predicted instrument tip location falls within the same receptive field as the ground truth location would, we expect that our findings can be replicated. Our dataset did not include images where the instrument tips were occluded. The sensitivity of our method to occlusions needs further study. In addition, we used two datasets drawn as convenience samples, which can affect the reproducibility of our findings in new datasets in which the videos systematically represent the population of surgeons. The domain shift in our work was in terms of expert and novice surgeons, our training dataset included videos of procedures by expert surgeons but our test dataset included only videos from novice surgeons. There may be other attributes to the domain shift, which we did not evaluate in this study. In a future study, to evaluate sensitivity to domain shifts, the sample of surgeons should be either exhaustive or representative to those in training and in practice, e.g., across levels of experience, gender, age, and other relevant attributes. In addition, the videos should capture procedures of different technical difficulties.

## Conclusion

VBA is a rapidly emerging application for surgeons to acquire and maintain skills. Our work advances algorithms for objective assessment of surgical skills. In networks used for VBA of surgical skill, supervising the spatial attention for image features with instrument tip trajectories improved model specificity by 10% and robustness against domain shifts (at least 32% for specificity). Using our methods to develop better algorithms with large datasets can provide surgeons with useful tools for improving skills and eventually improving patient outcomes.

## Data Availability

All data generated or analysed during this study are included in this published article.
